# Haematological, Biochemical and Hormonal Biomarkers of Heat Intolerance in Military Personnel

**DOI:** 10.3390/biology10101068

**Published:** 2021-10-19

**Authors:** Faith O. Alele, Bunmi S. Malau-Aduli, Aduli E. O. Malau-Aduli, Melissa J. Crowe

**Affiliations:** 1 College of Healthcare Sciences, James Cook University, Townsville QLD 4811, Australia; 2 College of Public Health, Medical and Veterinary Sciences, James Cook University, Townsville QLD 4811, Australia; aduli.malauaduli@jcu.edu.au; 3 College of Medicine and Dentistry, James Cook University, Townsville QLD 4811, Australia; bunmi.malauaduli@jcu.edu.au; 4 Division of Tropical Health and Medicine, James Cook University, Townsville QLD 4811, Australia; melissa.crowe@jcu.edu.au

**Keywords:** exertional heat illness, exertional heat stroke, heat tolerance, biomarkers, military

## Abstract

**Simple Summary:**

This study focuses on the biomarkers that are predictive of heat intolerance in military populations. Military personnel are at risk of exertional heat stroke when the body’s temperature increases during intense physical activity in hot weather. Exertional heat stroke (EHS) may accompany or precede heat intolerance, an unusual sensitivity to heat. However, it is unknown if blood biomarkers (haematological, biochemical and hormonal) are predictive of heat intolerance. We subjected a sample of Australian Defence Force personnel and civilian volunteers to a heat tolerance test (HTT), and blood samples were obtained pre-and post–HTT. The results showed that a history of EHS was associated with changes in creatinine and urea. The biochemical and hormonal biomarkers associated with heat intolerance were alanine amino transaminase, creatine kinase, cortisol and creatinine. Furthermore, creatinine and cortisol were identified as predictors and useful biomarkers of heat intolerance. This study also highlights the need for further exploration of genetic biomarkers to aid early identification and the return to duty process for military personnel who may be at risk of heat intolerance.

**Abstract:**

Heat intolerance is the inability to withstand heat stress and this may occur due to exertional heat stroke (EHS). However, it is unknown if heat intolerance is associated with immune and hormonal disturbances. This study investigates haematological, biochemical and hormonal biomarkers related to heat intolerance and EHS in military and civilian volunteers. A quasi-experimental pre-and post-test design was used, with participants drawn from the Australian Defence Force (ADF) and the general populace. Blood samples were collected and analysed for biomarkers. Inferential statistics compared the biomarkers between the groups. Changes in alanine amino transaminase (*p* = 0.034), creatine kinase (0.044), cortisol (*p* = 0.041) and creatinine (*p* < 0.001) differed between the heat-intolerant and heat-tolerant groups. Participants with a history of EHS showed significant changes in creatinine (*p* = 0.022) and urea (*p* = 0.0031) compared to those without EHS history. Predictors of heat intolerance were increasing post-HTT creatinine and cortisol (OR = 1.177, *p* = 0.011 and OR = 1.015, *p* = 0.003 respectively). Conclusively, EHS history is associated with changes in creatinine and urea concentrations, while the predictors of heat intolerance are creatinine and cortisol. However, further exploration of other biomarkers, such as genetic polymorphism, is needed.

## 1. Introduction

Australia has experienced an increase in ambient temperature by 1 °C since 1910, with heat waves projected to increase in the future [1]. The extreme heat conditions threaten the health and wellbeing of outdoor workers, including labourers, essential services workers, farmers, athletes and military personnel [2]. The military organisation responsible for defending Australian sovereignty and national interests is the Australian Defence Force (ADF) [3]. The physical activities performed by the ADF in regions characterised by extreme hot conditions increase the risk of exertional heat illness (EHI) [4]. EHI is a severity-based spectrum of disorders, and exertional heat stroke (EHS) is a severe form of EHI [5]. When EHS occurs, it is essential to determine if the personnel can return to duty [6]. The ADF return to duty involves using the standard heat tolerance test (HTT) developed by the Israeli Defence Force [7,8] to establish the heat tolerance status of the personnel [9]. Evidence from our recent study [10] showed that 56% of the ADF members who had previously experienced EHS and participated in the HTT were heat-intolerant. While the test is useful in determining heat tolerance status at the time of testing, the ability of the test to predict the reoccurrence of EHS is debatable [10,11]. Given the debate, there have been suggestions to include blood biomarker measurements in the HTT protocol [10,11]. This approach could increase the ability to identify military personnel who are unfit to return to duty [11]. While the effects of exercise in hot environments and EHI on immune and hormonal function have been documented [12,13,14], there is still limited evidence on the assessment of blood biomarkers associated with heat intolerance.

Engaging in physical activity in hot climates may cause perturbations in immune and hormonal functions [13]. Prolonged high-intensity exercise without sufficient recovery can cause transient immune disturbances [12]. The disturbances in immune function during intense exercise are similar to the responses elicited during EHS. The immune alterations include the suppression of immune cell concentration and increased leucocyte activity [14]. Evidence suggests that various factors may account for the immune responses during intense exercise in the heat [15]. These factors include elevated body temperature, stress hormones, leukocyte trafficking or their combination [15]. Studies investigating the effect of exercise in the heat on leukocytes showed that leukocytosis occurs when body temperature increases by >1 °C [16,17]. Elevated lymphocyte subsets (T suppressor, natural killer and total lymphocyte counts) have been reported in military recruits with EHI compared to those without EHI [18]. Changes in circulating lymphocytes may be explained by the rise in body temperature and the release of stress hormones such as cortisol [19].

Cortisol is an immune modulator secreted during exertion in scorching conditions as a result of increasing exercise intensity and rising core temperature [20]. A significant increase in cortisol levels was reported when core temperatures exceeded 39 ℃ during a heat tolerance assessment conducted among military volunteers [21]. Cortisol also induces the redistribution of circulating leukocyte subsets from the bone marrow during heat stress [20]. While leukocyte changes have been observed during physical exertion in hot environmental conditions, varying effects of heat exposure on other haematological parameters during physical activity have been reported. A study using blood samples obtained from laboratory personnel reported no significant changes in erythrocytes, haemoglobin concentration and mean corpuscular haemoglobin (MCH) during heat exposure [22]. However, marked changes in haematocrit levels and mean corpuscular volume (MCV) were observed [22]. In contrast, significantly reduced red blood cell (RBC) levels, packed cell volumes and platelet counts were observed among bakery workers exposed to heat [23]. However, in military populations, research is limited. More focus on military personnel is needed because of the possibility of extended high-intensity exercise in the heat, impacting their immune/hormonal responses and contributing to heatstroke symptoms [16]. In addition, heat stress and exercise in hot weather induces sweating, which may cause electrolyte and fluid imbalance, ultimately resulting in dehydration [24]. Dehydration occurs due to a lack of fluids or a mismatch between thirst and water requirements during exercise in the heat [25]. Moderate dehydration during exercise in the heat evokes significant increases in plasma cortisol [26]. Thus, maintaining water and electrolyte balance during exercise in the heat increases plasma volume and reduces osmolarity and cortisol levels [26].

Furthermore, during intense physical exertion, biochemical biomarkers of muscle and kidney function have been reported to be elevated post-exercise [27]. These biomarkers include alanine aminotransferase (ALT), aspartate aminotransferase (AST), creatine kinase (CK), creatinine and urea [27]. During physical activity, the increase in ALT, AST, CK and creatinine varies depending on the type, intensity and duration of the exercise [27]. These biomarkers have also been used to determine the severity of injury and organ damage that may occur in sequence to EHS [11]. Elevated CK, AST, ALT and creatinine levels in response to heatstroke indicate skeletal muscle and renal injury [28,29]. DuBose et al. reported that creatinine was elevated among military recruits following an EHI event and was indicative of muscle damage [18]. A recent review reported elevated levels of the biomarkers among military personnel who had experienced heat stroke. For example, mean CK levels were 6523.1 U/L (>200 times the average), ALT was 166.9 U/L (>20 times the average), and AST was 180.4 U/L (>10 times the average) [29]. Biomarkers have also been used to monitor clinical recovery from EHS [11]. Thus, these clinical biomarkers should be considered during HTT, given that the protocol is used to determine level of recovery before military personnel are allowed to return to duty.

Given that EHI may share similar immune disturbances to exercise, it is imperative to identify the biomarkers associated with the immune, biochemical and hormonal systems, understand the underlying mechanisms for their expression during the HTT and their predictive ability. While the current protocol identifies the heat tolerance state, it does not identify possible residual injury or underlying organ damage. The inclusion of potential clinical biomarkers (including haematological, biochemical and hormonal parameters) in the HTT protocol could aid early identification of individuals who are unfit to return to duty [6,11]. Furthermore, while previous studies among military personnel have focused on physiological factors associated with heat intolerance [10,30,31], little attention has been given to the effect of the HTT on military personnel’s haematological, biochemical and hormonal parameters.

Therefore, this study aims to determine if heat-intolerant individuals showed altered clinical biomarker responses (haematological, biochemical and hormonal) to the HTT compared to heat-tolerant individuals. We also seek to determine if ADF members with a history of EHS exhibited altered biomarker responses (haematological, biochemical and hormonal) to the HTT compared to individuals without a history of EHS. In addition, we aim to explore the biomarkers that predicted heat intolerance. First, we hypothesise that heat-intolerant individuals will have altered haematological, biochemical and hormonal responses to the HTT compared to their heat-tolerant counterparts. Second, we hypothesise that participants with a previous history of EHS will have altered haematological, biochemical and hormonal biomarkers compared to participants without a history of EHS. Third, we hypothesise that biochemical and hormonal biomarkers will predict heat intolerance, adjusted for age, gender and a history of EHS.

## 2. Materials and Methods

This study employed a quasi-experimental pre-and post-test design. It is part of a comprehensive study investigating the demographic, anthropometric [10], biochemical, haematological and genetic predictors of heat intolerance in military populations.

### 2.1. Participants

A minimum required sample size of 34 was determined *a priori* using G-power analysis. The statistical power was set at 80% to detect a medium-sized effect with a statistical significance level of 0.05. Participants were recruited from the military (*n* = 16), university (*n* = 22) and athletic community (*n* = 14). A medical screening form was used to obtain the medical history of participants in person or via emails. Written and verbal information about the study was provided, and written consent was obtained from all participants. Although we recruited ADF members to the study who had a previous history of EHS, we could not recruit controls from the ADF due to ethical issues linked to the possible career risks associated with heat intolerance. As part of the return to duty process [9], ADF members are referred for the HTT after a suspected or known episode of EHS (author MJC is a provider for heat tolerance testing to the ADF). Members (14 males and two females) referred for the HTT who volunteered for the study were recruited. While the exact timing of the HTT following EHS could not be controlled, ADF members were tested at least seven weeks or more after their EHS incident (the average time between heat illness event and HTT was approximately 17 weeks). The other participants who volunteered for the study were athletes (five males and nine females) and students and staff of James Cook University (19 males and three females). There were 11 triathletes and four (4) single sport (swimming and running) athletes (median years of training = 4.2) in the study. The athletes undertook an average of 4–7 training sessions per week of moderate to high intensity. In addition, the students and staff were a physically active group who engaged in 3–4 sessions per week of low to moderate-intensity exercise. Control participants and the ADF members were matched with regards to age, gender and aerobic capacity. The reasons for exclusion in the study were (1) a history of hypertension, malignant hyperthermia, or diabetes, (2) pregnant or lactating women, (3) undergoing treatment for anaemia, (4) undergoing treatment for a mental disorder and (5) using glucose-lowering agents or prednisolone or beta-blockers. Participants were advised to avoid heat exposure, exhaustive exercise, alcohol and caffeine for 24 h before taking part in the HTT. Full details of the methodology have been previously published [10].

### 2.2. Experimental Procedure

The HTT was performed at the same time of day, in the morning, to minimise any possible effects of circadian rhythms. Prior to commencing the test, the hydration status of the participants was determined using urine specific gravity (USG) (Atago handheld refractometer, Atago Co, Ltd., Itabashi, Tokyo, Japan). Participants were requested to arrive at the laboratory euhydrated (USG of <1.015). Participants with USGs > 1.015 were rehydrated with water, and a second assessment of their USG was performed to confirm their hydration status. Resting HR (Polar T31 Coded Transmitter, Polar Electro Oy, Kempele, Finland), blood pressure (Aneroid Sphygmomanometer Two-Handed, ALP-K2, Tokyo, Japan) and body weight (Tanita RD-545, Tanita Corporation, Tokyo, Japan) were measured. In addition, venous blood samples were collected from the participants before and after the HTT (see details below).

### 2.3. Heat Tolerance Test

The HTT was conducted in a climate control chamber set to 40 °C and 40% relative humidity (hot/dry conditions) [7]. Participants walked on a treadmill for 2 h at 5 km/h with a 2% incline. Heat intolerance was determined if the core temperature was >38.5 °C, the heart rate (HR) was >150 bpm or when the core temperature failed to plateau (increase > 0.45 °C during the 2nd hour of the HTT). The ADF uses the HTT criteria published by Dryan et al. [7]. The HTT was discontinued if the participants either experienced any symptoms of heat illness (including nausea, headaches, weakness, dizziness, etc.) or requested to stop the test. During the test, participants were provided with water ad libitum. The heat tolerance measures obtained during the test were rectal core temperature and heart rate. Rectal core temperature (RET-1 Rectal Probe, Physitemp Instruments, LLC, Clifton, NJ, United States) and heart rate were obtained at the start of the test and assessed every 5 min during the test.

### 2.4. Venous Blood Collection

Prior to and immediately after the HTT, 4 mL, venous blood samples were obtained from the anti-cubital fossa for haematological, biochemical and hormonal analyses while participants were sitting upright and resting. Blood was collected in EDTA (Ethylenediaminetetraacetic acid) tubes and analysed for white blood cell count, lymphocytes, monocytes and platelets using an automated haematology analyser (Coulter AcT diff Hematology Analyzer, Beckman Coulter, New South Wales, Australia). The biochemical and hormonal analyses samples were collected using a serum separator collection tube and centrifuged at 4500 rpm for 10 min for serum separation. The serum was frozen at −80 °C for subsequent sodium (Na), potassium (K), chloride (Cl), aspartate aminotransferase (AST), alanine aminotransferase (ALT), urea, creatine kinase (CK) and creatinine analyses (AU480 Chemistry Analyzer, Beckman Coulter, New South Wales, Australia). Hormonal analysis for serum cortisol utilised the Access reagent kit (Access 2 Immunoassay System, Beckman Coulter, New South Wales, Australia). The selected biomarkers are investigated after a heat stroke event and are useful indicators of injury severity and clinical recovery [11].

### 2.5. Statistical Analyses

Statistical analyses were conducted using the Statistical Package of Social Sciences (SPSS Statistics, Version 26, IBM Corp, Armonk, NY, USA) and the R Project for Statistical Computing (version 3.6.3). Heat tolerance status was analysed as a categorical dichotomous variable (heat tolerant versus heat intolerant). Final core temperature (Tc) and final heart rate (HR) as continuous variables were used to define heat tolerance. The measures compared between groups were complete blood count variables, Na, K, Cl, AST, ALT, urea, CK, creatinine and cortisol. The data were assessed for normality using the Shapiro–Wilk test. To determine the difference in pre-test and pre-test biomarkers between the groups (heat tolerant vs heat intolerant and EHS vs no history of EHS), Independent sample *t*-tests or the Mann–Whitney U test was conducted depending on the normality of the data. There were significant differences between the groups at pre-test for some measures. Thus, Mann––Whitney U tests were then used to identify the differences in measurements from pre-test to post-test between groups (heat tolerant vs. heat intolerant and EHS vs. no history of EHS). Correlations between heat tolerance outcomes (Tc and HR) and haematological, biochemical and hormonal markers were analysed using the Pearson correlation test.

A binary logistic regression model was used to assess and identify the influence of the post-HTT biomarkers on heat intolerance. All biochemical and hormonal measures were included in the logistic regression model, given that only some of the biochemical and hormonal measures changed between the groups (heat tolerance versus heat intolerance) over the time points of the HTT. The variables were entered into the model, non-significant variables were removed one at a time, and the impact on the remaining variables was assessed. If the odds ratios (ORs) changed more than 10%, the variable was retained in the model. Confounders adjusted for in the logistic regression were demographic characteristics (age and gender), EHS history, exercise intensity-defined using the rating of perceived exertion (RPE) and dehydration (% body mass loss). The level of significance was set at *p* < 0.05.

### 2.6. Ethics Approval

Written informed consent was obtained from all participants. The study was conducted in accordance with the Declaration of Helsinki and the National Health and Medical Research Council’s *National Statement on Ethical Conduct in Human Research*. Ethical approval was provided by the Departments of Defence and Veterans’ Affairs Human Research Committee (075-18) on 21 January 2019. 

## 3. Results

Fifty-two (52) volunteers participated in this study. The mean age of the study population was 31.1 ± 11.6 years, and 38 (73%) were males. Participants who had a previous history of EHS accounted for 31% (n = 16) of the population. Overall, 23 (44.2%) participants were categorised as heat intolerant based on the HTT criteria. Among the participants with a history of EHS, nine (56%) were classified as heat intolerant. A summary of the previously reported physiological and anthropometric findings has been included to provide more context for the results of this study (Table S1) [10]. The physiological and anthropometric parameters indicated that the heat-intolerant participants had significantly higher body weight (85.3 vs. 75.5, *p* = 0.007), BMI (27 vs. 24.4 *p* = 0.011), BF% (24.1 vs. 20.2, *p* = 0.034) and BSA (2.0 vs. 1.9, *p* = 0.017). On the other hand, the heat-intolerant participants had lower BSA/M _ratio_ (240.4 vs. 256.5, *p* = 0.005) and VO_2_max (39.2 vs. 48.2, *p* < 0.001). In addition, the heat-intolerant group had significantly higher final T_c_, (38.67 vs. 37.96, *p* < 0.001), final HR (154 vs. 122, *p* < 0.001) and RPE (13 vs. 11, *p* = 0.016). However, there was no significant difference in sweat rate (1.32 vs. 1.18, *p* = 0.399) and percentage dehydration (% BM loss) between the groups (1.53 vs. 1.38, *p* = 0.645).

### 3.1. Differences in Haematological and Biochemical Variables between Heat-Tolerant and Intolerant Participants

Table 1 displays all haematological measures pre-test, post-test and the differences between pre-and post-test for the heat-tolerant and heat-intolerant groups. No significant differences were observed between the heat-tolerant and heat-intolerant groups for any of the haematological measures at pre-test. Similarly, there was no significant difference between the groups for all the haematological measures when the differences pre- to post-test were examined (Table 1 and Figure S1).

In Table 2, a significant difference in ALT at pre-test was observed with the heat intolerant having a higher ALT level than their heat-tolerant counterparts. No other biochemical or hormonal measure differed at pre-test. However, a significant effect was observed between the groups for the pre-and post-test differences between the heat-tolerant and heat-intolerant groups (Table 2 and Figure S1). The change in ALT, CK and creatinine from pre-test to post-test was significantly different between the heat-tolerant and heat-intolerant groups. ALT, CK and creatinine show a more significant increase in the heat-intolerant group compared to the heat-tolerant group *p* = 0.034, *p* = 0.044 and *p* < 0.001, respectively). In contrast, cortisol concentration for the heat-tolerant group decreased from pre-test values after the HTT, while the concentration for the heat-intolerant group increased (*p* = 0.041).

### 3.2. Differences in Haematological and Biochemical Measures between Participants with a History of EHS and Participants without a History of EHS

Table 3 shows the differences in haematological measures for participants with and without a history of EHS. Pre-test, there was a significant difference in haemoglobin concentration (*p* = 0.026) and granulocyte count (*p* = 0.040) between participants with a history of EHS and those without EHS history. However, there was no significant difference between the groups when the pre- to post-test differences were examined (Table 3 and Figure S2).

In Table 4, there were no significant differences in biochemical and hormonal measures between the groups pre-test. However, the change in creatinine and urea from pre- to post-test was significantly different between the groups (Table 4 and Figure S2). The increase in creatinine concentration from pre- to post-test was higher among participants with a history of EHS compared to those without EHS history (*p* = 0.022). Furthermore, participants with a history of EHS showed an increase in urea concentration after the HTT, whereas the group without a history of EHS showed a decrease in urea after the HTT (*p* = 0.0031).

### 3.3. Correlations between Haematological, Biochemical Measures, Core Temperature and Heart Rate

**Figure 1:**
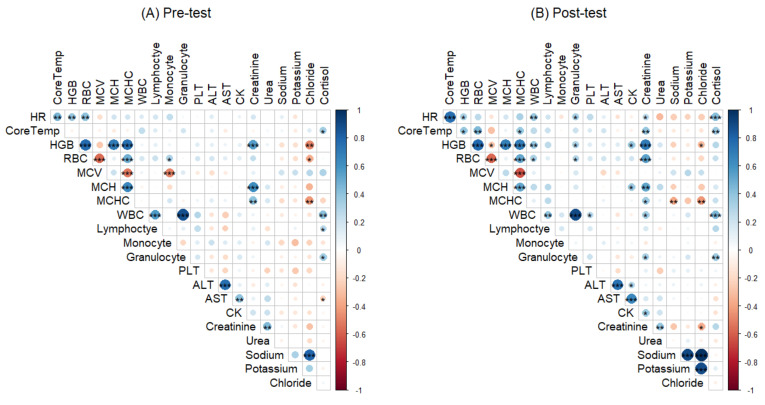
Correlations between heat tolerance outcomes (Tc and HR) and the haematological, biochemical and hormonal biomarkers (**A**) pre-test and (**B**) post-test. Significant * *p* < 0.05, ** *p* < 0.01, *** *p* < 0.001; WBC: White blood cell; RBC: Red blood cell; MCV: Mean corpuscular volume; MCH: Mean corpuscular haemoglobin; MCHC: Mean corpuscular haemoglobin concentration; CK: creatine kinase; ALT: alanine transaminase; AST: aspartate transaminase. Dark blue to dark red colour indicates Pearson’s correlation coefficient from 1 to −1, respectively.

Correlations between the heat tolerance outcomes (core temperature and heart rate) and haematological, biochemical and hormonal variables were assessed (Figure 1). Baseline biomarkers correlated with core temperature included cortisol (R = 0.320, *p* = 0.025) while haemoglobin (R = 0.363, *p* = 0.009) and RBC (R = 0.370, *p* = 0.008) were correlated with heart rate. Post-test biomarkers significantly correlated with core temperature were haemoglobin (R = 0.340, *p* = 0.015), RBC (R = 0.436, *p* = 0.001), MCHC (R = 0.277, *p* = 0.049), creatinine (R = 0.439, *p* = 0.001) and cortisol (R = 0.367, *p* = 0.007). Heart rate was significantly correlated with haemoglobin (R = 0.282, *p* = 0.045), WBC (R = 0.362, *p* = 0.009), creatinine (R = 0.274, *p* = 0.049) and cortisol (R = 0.451, *p* < 0.001).

The predictive relationship between biochemical and hormonal biomarkers and heat intolerance was assessed via a binary logistic regression adjusting for age, gender and EHS history, %BM loss and RPE (Table 5). The findings show that with increasing creatinine and cortisol level, participants were approximately 1.18 and 1.02 times more likely to be heat intolerant (OR = 1.177, 95% CI 1.039–1.333, *p* = 0.011 and OR = 1.015, 95% CI 1.005–1.025, *p* = 0.003 respectively).

## 4. Discussion

Exposure to heat and physical activity can affect and modify an individual’s immune function and blood parameters [22,32]. Moderate levels of heat exposure or light physical activities stimulate immune responses [32]. On the other hand, severe heat exposure and exhausting physical activity have a suppressant effect causing a temporary increase in susceptibility to infections and EHS [32]. Given that EHS may precede or accompany heat intolerance, it is essential to identify the biomarkers predictive of heat intolerance [14,33]. In the current study, there are significant differences in the baseline and post-test values for ALT, creatine kinase, creatinine and cortisol among the heat-intolerant group compared to the heat-tolerant group. When the predictive role of the biochemical biomarkers is assessed, the biomarkers found to be predictive of heat intolerance were creatinine and cortisol.

While the heat tolerant participants had lower plasma cortisol levels post-test, their heat-intolerant counterparts had increased levels post-test, though the values were within normal clinical limits. These findings are similar to previous studies that have reported minimal change in circulating cortisol levels during either light to moderate exercise, when the core temperature is clamped or when the exercise is interrupted [34,35,36]. Therefore, this indicates that cortisol level is more likely to be markedly increased during heavy prolonged continuous exercise [35]. After a live-fire training, higher post-test cortisol levels have been reported among firefighters in previous studies [37]. Cortisol levels during exercise may also be linked to exercise intensity and dehydration [38,39]. According to Caetano et al., exercise intensity and high cortisol levels were correlated among rugby players [38]. Similarly, Castro–Sepulveda et al. reported elevated post-game cortisol levels among soccer players who were mildly dehydrated before commencing the game [39]. However, it is essential to note that rugby and soccer games are high-intensity exercises. Our study showed that the rating of perceived exertion and dehydration were not predictive of heat intolerance. The HTT is low intensity, and we ensured that our participants were well hydrated. Nonetheless, the effect of dehydration and exercise intensity during the HTT should be explored. Exercising in high ambient temperatures has a synergistic effect on the exercise stress response, and elevated cortisol signifies the body’s response to stress [40]. There are plausible explanations for the observed relationship between heat intolerance and cortisol in our study. First, fitness levels and training have been shown to reduce catabolic responses to exercise-induced stress [41]. The increased cortisol level in the heat-intolerant group may be a reflection of a more significant physiological stress due to lower fitness levels [41,42]. Evidence from our previous study showed that the VO2max of heat-intolerant participants was significantly lower (39.2 mL/kg/min vs. 48.2 mL/kg/min) than that of the heat-tolerant group [10]. In addition, approximately 48% of the heat-tolerant group were highly fit athletes with rigorous training schedules that may have contributed to the lower cortisol levels. Second, heat acclimation may play a role in reducing the hormonal responses to heat stress [41]. However, physiological adaptations during heat acclimatisation or acclimation are similar to adaptations to aerobic training. Therefore, well-trained or fit individuals are more likely to show cardiovascular adaptations similar to those of heat-acclimatised individuals, which may have augmented the reduced cortisol response in the heat tolerant group [29]. High cortisol levels may increase risk-taking behaviour and affect decision-making processes [36,43]. The effect of cortisol is vital in military settings where exposure to heat during activities may impose significant psychological and physiological strain resulting in illness and death from EHS [21]. Given that cortisol is amplified during exercise and heat stress, cortisol’s potential utility in surveillance for heat intolerance in military settings should be considered [21].

Other biochemical markers, including ALT, CK and creatinine, changed following the heat tolerance test between the heat-tolerant and heat-intolerant groups. However, of these biomarkers, only creatinine was predictive of heat intolerance. ALT and CK are considered as markers for liver injury and muscular damage, respectively [44]. In our study, ALT was higher in the heat intolerant group at pre-test, and the change over time differed between the heat-intolerant and heat-tolerant groups. Although the pre-test values were different between the groups, this finding should be interpreted with caution, given that both the pre-test and post-test values were within the normal range. According to published evidence, marked increases in ALT concentrations are dependent on the intensity and duration of the exercise [44,45]. The studies reported that markedly elevated ALT levels were observed after strenuous and prolonged activities such as ultramarathons in athletes [44,45]. In our study, the exercise was low intensity and did not show elevated ALT levels above clinical limits. However, the heat intolerant group may have been exerting themselves more than the heat-tolerant group. The heat-intolerant participants had a higher physiological strain index (5.6 vs. 3.5) and reported higher ratings of perceived exertion (13 vs. 11) during the HTT compared to their heat-tolerant counterparts [10].

Furthermore, heat exposure has been shown to cause a significant increase in ALT [46]. There was a higher change in ALT in our study following heat exposure in the heat-intolerant group. A study conducted among rats during a heat stress test showed that the rats who had whole-body heat exposure had higher ALT concentrations and showed extensive liver damage than those without heat exposure [46]. Similarly, the change in CK levels varied between the groups. The heat intolerant showed a greater change in CK concentrations compared to the heat-tolerant group. Evidence suggests that increased CK concentration may reflect the intensity of the exercise [47]. The concentration of CK in our study was within the normal clinical limit and may reflect the low-intensity exercise. Thus, it is plausible that if participants are exposed to high-intensity physical activity in hot conditions, the level of CK may increase significantly in those who are heat intolerant [45]. House et al. reported CK increased post-test for three groups of participants during an HTT (participants with a history of EHI, controls and those susceptible to malignant hyperthermia). However, no significant difference in CK levels between the groups of participants was observed [48]. Our study differed from the cited study, given that we compared CK levels between the heat-tolerant and heat-intolerant groups [48]. Our study findings may reflect the physiological strain and higher work intensity of the heat-intolerant participants, given that they had lower aerobic capacity/fitness levels [10]. It has been reported that significant increases of CK after exercise are usually lower in trained subjects compared to untrained subjects [49,50]. The current study’s findings are important for military populations as ALT and CK could be helpful markers for monitoring recovery after EHS and evaluating physical and heat strain during training [51,52]. However, this finding needs to be interpreted with caution and replicated in a larger study.

The findings of this study show that increasing levels of creatinine are predictive of heat intolerance. Current evidence suggests that creatinine is a clinical biomarker of kidney function and elevated creatinine levels indicate acute kidney injury [53]. Pryor et al. reported that creatinine levels increased during exercise in hot conditions and reached the threshold for acute kidney injury [53]. The authors attributed the increase in creatinine levels to reducing plasma volume following dehydration [53]. Our study shows that dehydration is not predictive of heat intolerance; our participants were provided with water ad libitum throughout the exercise. The published evidence from our previous study also showed no significant difference in the level of dehydration between the heat-tolerant and intolerant participants [10]. The significant difference in creatinine values between the groups may not indicate altered renal function, given that the values were within the normal range. Instead, our study’s findings could be considered a possible indicator of increased risk of renal injury if the participants were to be exposed to hotter environmental conditions and more intense exercise [54]. While there is no predictive effect of exercise intensity on heat intolerance in this current study, exercise intensity may have an impact on creatinine. Evidence suggests that the type and intensity of the exercise might cause an increase in creatinine levels [55]. However, the effect of relative exercise intensity on creatinine during the HTT deserves further investigation.

Although a history of EHS was not predictive of heat intolerance, a significant difference in the biochemical biomarkers is observed between participants who had a previous history of EHS and those without EHS history. Changes in urea and creatinine are significantly different between the groups. It is possible that the change in creatinine and urea may indicate either residual injury or recovery. Given that a history of EHS may lead to a reoccurrence and creatinine is a predictor of heat intolerance, the creatinine values, while not alarming, may offer prime information regarding injury status [11].

### 4.1. Limitations

Despite being the first study to investigate biomarkers predicting heat intolerance in military personnel, this study has limitations. While we explored a range of haematological and biochemical biomarkers, other factors beyond the scope of this study may be expressed during the HTT. Owing to logistical difficulties, we only assessed the biomarkers pre- and post-HTT. Assessing the biomarkers during the test at peak core temperature could be considered in future research to provide further information about the rate of rise of the biomarkers and their predictive role in heat intolerance. Given the ethical issues associated with recruiting control from the ADF, our control group were recruited from the general populace. The control participants were matched to cases regarding gender, age and aerobic capacity. Although we tried to control the effect of alcohol and caffeine by asking participants to refrain from them before the HTT, we did not control for the impact of other dietary and nutritional supplements. Furthermore, given the small sample of females (n = 14), it was difficult to assess the effect of the menstrual cycle on female participants’ haematological, biochemical and hormonal responses. The role of the menstrual cycle could be considered in future research with a larger sample of female participants.

### 4.2. Implications for Policy and Future Research

The HTT is a valuable tool that aids the return to duty process, and our study has highlighted potential biomarkers that may be included as part of the return to duty screening process, including creatinine and cortisol. Given that the HTT does not account for organ damage after an EHS event, the inclusion of these biomarkers could possibly capture those who are not ready to return to duty [6,11]. While it is possible that the biomarkers cannot determine a reoccurrence of EHI or EHS, they could also help to determine recovery to some extent. However, they are useful indicators that could predict or provide information about who is more likely to be heat intolerant. The predictive relationship between heat intolerance, creatinine and cortisol in this current study is important and may play a significant role in determining return to duty. These results are potentially important for military personnel who are subjected to physical and psychological stress during EHS [56]. However, the study also highlights the need for further exploration of other biomarkers such as genetic biomarkers, which include heat shock proteins, and could identify heat intolerance. Heat shock proteins play a crucial role in thermotolerance, cellular stress response and exert an immunomodulatory effect during exercise [57]. While exertion in hot conditions has been reported to induce HSPs, little is known about the role of genetic polymorphisms in heat intolerance [58]. Therefore, it is important for future studies to explore the role of HSPs in heat intolerance. Exploring the genetic biomarkers may also enhance a better understanding of the mechanism of heat intolerance. In turn, understanding the mechanism can facilitate efforts to address current policies related to EHS, heat intolerance and return to duty in military and non-military settings.

## 5. Conclusions

In conclusion, heat intolerance is associated with changes in ALT, CK, creatinine and cortisol concentrations. In addition, a history of EHS was associated with significant changes in creatinine and urea levels. Evidence from this study shows that increasing creatinine and cortisol increased the risk of heat intolerance. However, further research is needed to identify other potential biomarkers, such as genetic markers, that will aid the identification of those at risk of heat intolerance and aid the return to duty process. 

## Figures and Tables

**Table 1:** Differences in haematological measures between heat-tolerant and intolerant groups.

Variables	RV ^‡^	Group	Pre-Test	Post-Test	∆ from Pre-Test to Post-Test ^†^
Haemoglobin (g/L) *	110–180	Heat tolerant	134.10 (12.82)	134.82 (14.80)	0.50 (3.50)
		Heat intolerant	140.00 (13.13)	142.17 (14.47)	2.00 (11.00)
RBC × 10^12^/L *	4.0–6.0	Heat tolerant	4.59 (0.35)	4.67 (0.38)	0.065 (0.12)
		Heat intolerant	4.73 (0.28)	4.88 (0.33)	0.14 (0.38)
MCV (fL) *	80.0–99.9	Heat tolerant	92.59 (5.46)	92.06 (5.89)	−0.35 (1.92)
		Heat intolerant	92.57 (5.03)	91.47 (5.69)	−0.60 (1.35)
MCH (pg) *	27.0–31.0	Heat tolerant	29. 27 (1.64)	28.92 (1.89)	−0.20 (0.88)
		Heat intolerant	29.58 (1.77)	29.12 (1.95)	−0.20 (0.72)
MCHC (g/L) *	330.0–370.0	Heat tolerant	316.69 (22.47)	313.46 (23.95)	−1.00 (10.25)
		Heat intolerant	317.72 (28.88)	320.43 (25.92)	1.00 (12.25)
WBC × 10^9^/L*	4.5–10.5	Heat tolerant	5.40 (1.26)	6.74 (1.70)	0.95 (1.74)
		Heat intolerant	5.96 (1.15)	7.71 (1.58)	1.88 (1.84)
Lymphocytes × 10^9^/L ^†^	1.2–3.4	Heat tolerant	1.70 (0.62)	1.84 (0.50)	0.25 (0.44)
		Heat intolerant	1.77 (0.83)	1.77 (0.92)	0.20 (0.54)
Monocytes × 10^9^/L ^†^	0.1–0.6	Heat tolerant	0.39 (0.18)	0.45 (0.19)	0.03 (0.23)
		Heat intolerant	0.37 (0.18)	0.44 (0.23)	0.10 (0.24)
Granulocytes × 10^9^/L*	1.4–6.5	Heat tolerant	3.25 (1.00)	4.37 (1.42)	0.95 (1.46)
		Heat intolerant	3.65 (1.07)	5.30 (1.69)	1.29 (2.09)
Platelets × 10^9^/L*	150–450	Heat tolerant	232.45 (69.03)	256.36 (78.92)	19.50 (30.75)
		Heat intolerant	235.36 (74.53)	262.55 (86.46)	24.00 (33.00)

RV: Reference value; ^†^ Mann–Whitney U test was used (medians and interquartile range of the raw data are reported); * Independent samples *t*-test was used (mean and SD of the raw data are reported); WBC: White blood cell; RBC: Red blood cell; MCV: Mean corpuscular volume; MCH: Mean corpuscular haemoglobin; MCHC: Mean corpuscular haemoglobin concentration; ∆: change. ^‡^ Reference values from the Coulter AcT diff Hematology Analyser

**Table 2:** Differences in biochemical and hormonal variables between the heat-tolerant and heat-intolerant groups.

Variables	RV ^‡^	Group	Pre-Test	Post-Test	∆ from Pre-Test to Post-Test ^†^
ALT (U/L) ^†^	7–52	Heat tolerant	18.80 (10.10) ^a^	20.25 (11.60)	0.80 (1.55)
		Heat intolerant	24.60 (11.00)	26.70 (12.90)	1.90 (1.95) ^b^
AST (U/L) ^†^	13–39	Heat tolerant	25.10 (13.10)	24.70 (13.40)	2.00 (3.13)
		Heat intolerant	26.80 (9.30)	28.00 (11.30)	3.25 (2.75)
CK (U/L) ^†^	30–223	Heat tolerant	140.35 (109.80)	142.00 (121.20)	12.75 (27.82)
		Heat intolerant	145.50 (141.00)	164.85 (122.50)	27.95 (24.50) ^b^
Sodium (mEq/L) ^†^	136–145	Heat tolerant	138.72 (2.70)	138.40 (3.00)	0.45 (3.02)
		Heat intolerant	137.60 (2.10)	137.70 (4.20)	−0.40 (5.50)
Potassium (mEq/L) ^†^	3.5–5.1	Heat tolerant	4.82 (2.00)	4.31 (0.28)	−0.10 (0.33)
		Heat intolerant	4.33 (0.29)	4.36 (0.54)	0.07 (0.56)
Chloride (mEq/L) ^†^	98–107	Heat tolerant	103.15 (3.50)	102.50 (3.00)	−0.45 (2.47)
		Heat intolerant	101.90 (3.30)	99.60 (7.40)	−2.05 (6.42)
Cortisol (nmol/L) *	193–690	Heat tolerant	341.37 (132.04)	225.69(104.11)	−108.44 (186.29) ^b^
		Heat intolerant	384.63(178.96)	391.07 (182.51)	5.07 (333.19)
Creatinine (µmol/L) *	53–110	Heat tolerant	74.11 (11.93)	78.28 (11.59)	5.00 (5.50)
		Heat intolerant	75.41 (13.65)	91.00 (13.85)	13.00 (11.75) ^b^
Urea (mmol/L) *	2.50–8.93	Heat tolerant	5.65 (1.30)	5.61 (1.21)	−0.07 (0.42)
		Heat intolerant	5.62 (1.57)	5.65 (1.48)	0.05 (0.68)

^†^ Mann–Whitney U test was used (medians and interquartile range of the raw data are reported); * Independent samples *t*-test was used (mean and SD of the raw data are reported); RV: Reference value; CK: creatine kinase; ALT: alanine transaminase; AST: aspartate transaminase; ∆: change. ^‡^ Reference values from AU480 Chemistry Analyzer, Beckman Coulter instructions for use. ^a^ significant difference (*p* < 0.05) between heat-tolerant and heat-intolerant groups at pre-test; b- significant difference (*p* < 0.05) in change from pre- to post-test between heat-tolerant and heat-intolerant groups.

**Table 3:** Differences in haematological variables between participants with and without a history of EHS.

Variables	RV ^‡^	Group	Pre-Test	Post-Test	∆ from Pre-Test to Post-Test ^†^
Haemoglobin (g/L) *	110–180	History of EHS	142.93 (14.23) ^a^	145.81 (15.55)	2.00 (9.00)
		No history of EHS	134.03 (11.93)	134.63 (13.50)	1.00 (3.00)
RBC × 10^12^/L *	4.0–6.0	History of EHS	4.75 (0.29)	4..91 (0.34)	0.09 (0.69)
		No history of EHS	4.61 (0.33)	4.70 (0.37)	0.06 (0.16)
MCV (fL) *	80.0–99.9	History of EHS	92.71 (5.54)	91.74 (5.92)	−0.80 (1.85)
		No history of EHS	92.53 (5.18)	91.82 (5.76)	−0.40 (1.45)
MCH (pg) *	27.0–31.0	History of EHS	30.00 (2.00)	30.00 (2.20)	−2.00 (0.90)
		No history of EHS	29.00 (1.50)	29.00 (1.70)	−2.00 (0.60)
MCHC (g/L) *	330–370	History of EHS	325.33 (24.15)	321.38 (23.48)	2.00 (18.50)
		No history of EHS	313.72 (25.11)	314.42 (25.49)	−1.00 (9.00)
WBC × 10^9^/L *	4.5–10.5	History of EHS	6.00 (1.07)	8.10 (1.59)	1.90 (1.19)
		No history of EHS	5.49 (1.28)	6.75 (1.60)	0.75 (2.56)
Lymphocytes × 10^9^/L ^†^	1.2–3.4	History of EHS	1.57 (0.90)	1.82 (0.95)	0.25 (0.40)
		No history of EHS	1.64 (0.45)	1.84 (0.73)	0.17 (0.57)
Monocytes × 10^9^/L *	0.1–0.6	History of EHS	0.33 (0.21)	0.50 (0.44)	0.10 (0.29)
		No history of EHS	0.40 (0.17)	0.44 (0.15)	0.03 (0.22)
Granulocytes × 10^9^/L *	1.4–6.5	History of EHS	3.89 (0.99) ^a^	5.62 (1.54)	1.47 (1.54)
		No history of EHS	3.23 (1.02)	4.41 (1.50)	0.87 (1.85)
Platelets × 10^/L *	150–450	History of EHS	267.93 (96.55)	300.93 (105.73)	27.00 (38.50)
		No history of EHS	219.44 (52.04)	241.14 (62.21)	19.00 (23.50)

^†^ Mann–Whitney U test was used (medians and interquartile range of the raw data are reported); * Independent samples *t*-test was used (mean and SD of the raw data are reported); WBC: White blood cell; RBC: Red blood cell; MCV: Mean corpuscular volume; MCH: Mean corpuscular haemoglobin; MCHC: Mean corpuscular haemoglobin concentration; ∆: change. ^a^ significant difference (*p* < 0.05) between heat-tolerant and heat-intolerant groups at pre-test; ^‡^ Reference values from the Coulter AcT diff Hematology Analyser.

**Table 4:** Differences in biochemical and hormonal variables between participants with and without a history of EHS.

Variables	RV ^‡^	Group	Pre-Test	Post-Test	∆ from Pre-Test to Post-Test ^†^
ALT (U/L) ^†^	7–52	History of EHS	24.45 (16.00)	26.55 (16.70)	1.90 (2.45)
		No history of EHS	22.20 (12.80)	23.50 (14.00)	0.80 (1.90)
AST (U/L) ^†^	13–39	History of EHS	28.85 (12.30)	31.90 (15.20)	3.20 (4.40)
		No history of EHS	23.45 (9.30)	24.80 (13.40)	2.20 (3.20)
CK (U/L) ^†^	30–223	History of EHS	163.55 (172.80)	176.35 (219.30)	15.10 (26.85)
		No history of EHS	140.35 (96.70)	153.50 (106.80)	20.40 (27.10)
Sodium (mEq/L) *	136–145	History of EHS	137.71 (1.62)	137.99 (2.46)	−0.40 (2.80)
		No history of EHS	138.13 (3.82)	133.99 (21.84)	0.40 (3.95)
Potassium (mEq/L) *	3.5–5.1	History of EHS	4.43 (0.34)	4.46 (0.31)	0.14 (0.40)
		No history of EHS	4.68 (1.78)	4.20 (0.72)	−0.10 (0.33)
Chloride (mEq/L) *	98–107	History of EHS	102.44 (3.39)	101.36 (3.91)	−1.00 (2.60)
		No history of EHS	103.38 (3.30)	99.10 (16.33)	−0.70 (3.80)
Cortisol (nmol/L) *	193–690	History of EHS	385.06 (172.44)	340.30 (214.10)	−25.73 (364.65)
		No history of EHS	351.08 (148.57)	280.42 (137.30)	−122.84 (195.47)
Creatinine (µmol/L) *	53–110	History of EHS	76.64 (13.00)	90.50 (15.05)	13.00 (17.50) ^b^
		No history of EHS	73.92 (12.54)	80.28 (11.966)	6.00 (4.50)
Urea (mmol/L) *	2.50–8.93	History of EHS	5.38 (1.14)	5.59 (1.10)	0.19 (0.68) ^b^
		No history of EHS	5.73 (1.51)	5.64 (1.42)	−0.14 (0.49)

^†^ Mann–Whitney U test was used (medians and interquartile range of the raw data are reported); * Independent samples *t*-test was used (mean and SD of the raw data are reported); RV: Reference value; CK: creatine kinase; ALT: alanine transaminase; AST: aspartate transaminase; ∆: change. ^‡^ Reference values from AU480 Chemistry Analyzer, Beckman Coulter instructions for use. ^b^ significant difference (*p* < 0.05) in change from pre- to post-test between heat-tolerant and heat-intolerant groups.

**Table 5:** Predictive relationship between post-test biochemical and hormonal variables and heat intolerance.

Variables	B	S.E.	Wald	df	*p* Value	OR	95% CI	
ALT	0.026	0.018	2.238	1	0.135	1.037	0.992	1.063
Creatinine	0.149	0.065	5.152	1	0.023	1.160	1.020	1.319
Sodium	0.084	0.086	0.954	1	0.329	1.088	0.919	1.287
Potassium	−2.872	2.253	1.625	1	0.202	0.057	0.001	4.681
Cortisol	0.015	0.006	7.185	1	0.007	1.015	1.004	1.026
Gender (Ref: Female)	−2.192	1.573	1.942	1	0.163	0.112	0.005	2.436
Age	−0.027	0.052	0.265	1	0.607	0.974	0.880	1.078
EHS (Ref: No history of EHS)	0.684	1.184	0.0334	1	0.563	1.983	0.195	20.175
%BM Loss	−0.064	0.472	0.019	1	0.892	0.938	0.372	2.365
RPE	0.236	0.201	1.378	1	0.240	1.266	0.854	1.877

ALT: alanine transaminase; EHS: Exertional heat stroke; %BM loss (per cent body mass loss); RPE (Rating of perceived exertion); CI: Confidence Interval; OR: Odds ratio; df: Degree of freedom; S.E: Standard error.

## Data Availability

The dataset supporting the conclusions of this article is included within the article and its additional files.

## Supplementary Materials

The following are available online at https://www.mdpi.com/article/10.3390/biology10101068/s1, Figure S1: Haematological and biochemical differences between the time points of the heat tolerance test and the heat-tolerant and intolerant participants, Figure S2: Haematological and biochemical differences between the time points of the heat tolerance test and the participants with and without a history of EHS, Table S1: Anthropometric characteristics and physiological responses of the heat-tolerant and heat-intolerant participants.
