# Supplementary material for: Haematological, Biochemical and Hormonal Biomarkers of Heat Intolerance in Military Personnel
Source: Biology (Basel). 2021 Oct 19;10(10):1068. doi: 10.3390/biology10101068 (PMC8533107; doi:10.3390/biology10101068)
Supplement: Supplementary file 1 [file biology-10-01068-s001.zip › biology-1388639-supplementary.pdf]

Table S1: Anthropometric characteristics and physiological responses of the heat tolerant and heat intolerant participants.

Characteristics	Heat Tolerant	Heat Intolerant	*p*-Value
Weight (kg) *	75.5 ± 12.10	85.3 ± 12.8	0.007
BMI (kg/m^2) *	24.4 ± 3.3	27.0 ± 3.9	0.011
BSA (m^2) *	1.9 ± 0.2	2.0 ± 0.2	0.017
BSA/M_{ratio} (cm^{-2}/kg) *	256.5 ± 20.0	240.4 ± 19.5	0.005
VO_2max (mL/kg/min) *	48.2 ± 10.5	39.2 ± 6.4	0.001
Body fat (%)*	20.2 ± 5.9	24.1 ± 7.0	0.034
Baseline T_C (°C) *	36.87 ± 0.29	37.02 ± 0.29	0.076
Final T_C (°C) *	37.96 ± 0.37	38.67 ± 0.22	<0.001
Baseline HR (bpm) *	65 ± 11	70 ± 10	0.109
Final HR (bpm) *	122 ± 18	154 ± 15	<0.001
Final RPE†	11.00 (3.0)	13 (6.0)	0.016
SR (L/hr) †	1.18 (0.28)	1.32 (0.80)	0.399
%BM loss (kg) †	1.38 ± 0.82	1.53 ± 1.59	0.645

BMI, body mass index; BSA/M ratio, body surface area to mass ratio; † Mann Whitney U test was used (medians and interquartile range of the raw data are reported); *Independent samples t-test was used (mean and SD of the raw data are reported). The table was adapted and reprinted from Alele et al [10]

10. Alele, F.O.; Malau-Aduli, B.S.; Malau-Aduli, A.E.O.; Crowe, M.J. Individual Anthropometric, Aerobic Capacity and Demographic Characteristics as Predictors of Heat Intolerance in Military Populations. *Medicina* **2021**, *57*, 173.

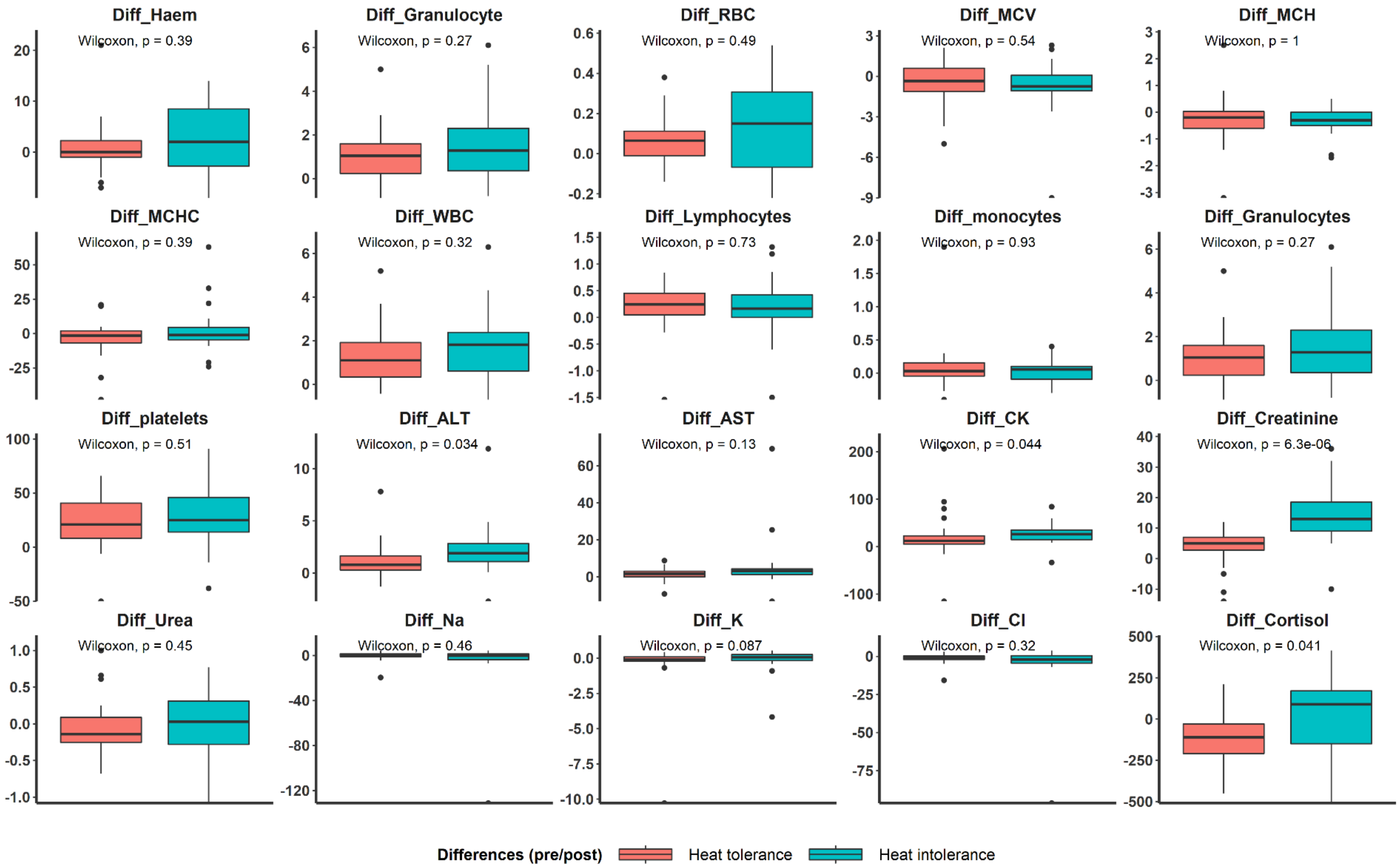

WBC: White blood cell; RBC: Red blood cell; MCV: Mean corpuscular volume; MCH: Mean corpuscular haemoglobin; MCHC: Mean corpuscular haemoglobin concentration; CK: creatine kinase; ALT: alanine transaminase; AST: aspartate transaminase; K: Potassium, Na: Sodium; Cl: Chloride

Figure S1: Haematological and biochemical differences between the time points of the heat tolerance test and the heat tolerant and intolerant participants

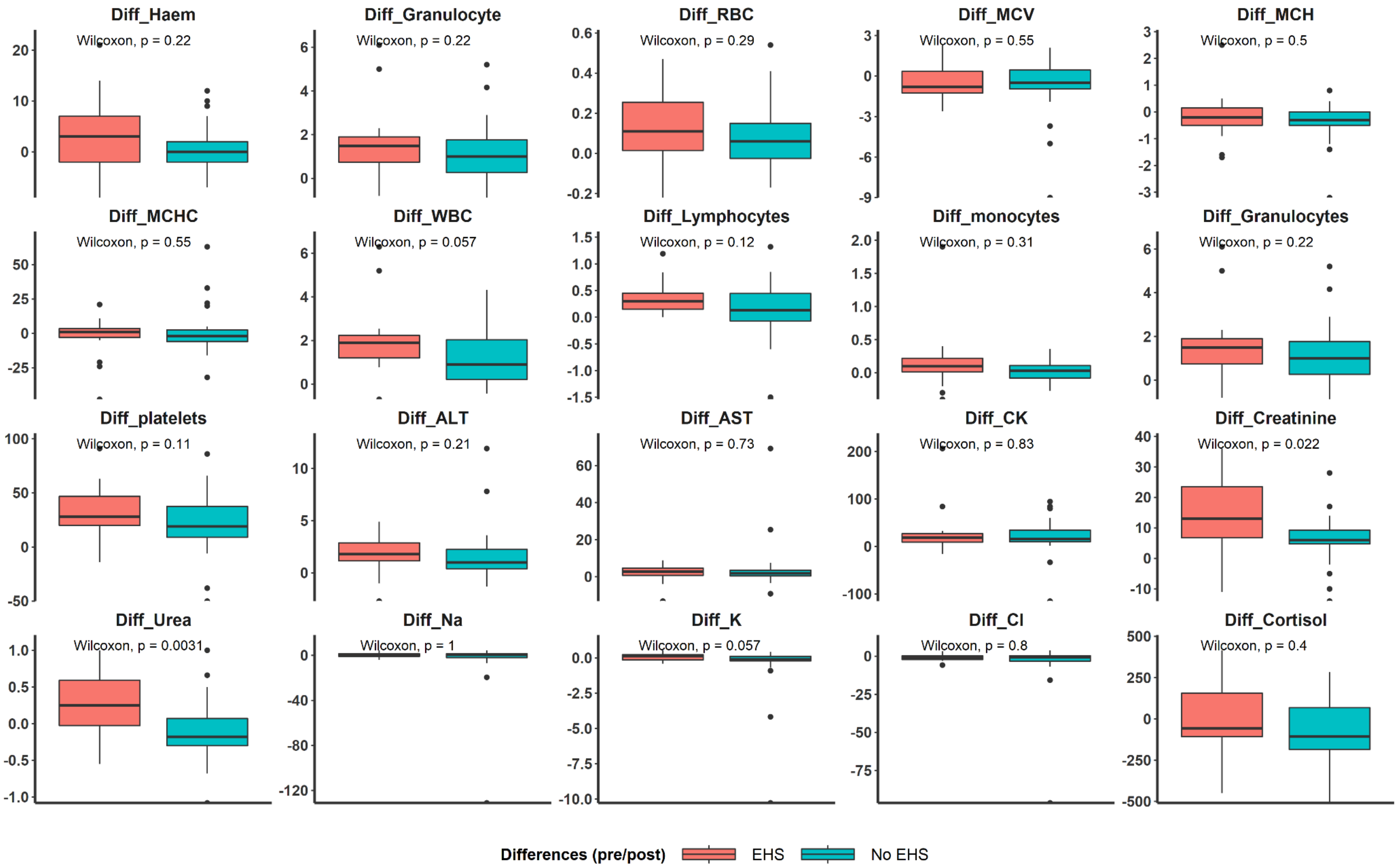

WBC: White blood cell; RBC: Red blood cell; MCV: Mean corpuscular volume; MCH: Mean corpuscular haemoglobin; MCHC: Mean corpuscular haemoglobin concentration; CK: creatine kinase; ALT: alanine transaminase; AST: aspartate transaminase; K: Potassium, Na: Sodium; Cl: Chloride

Figure S2: Haematological and biochemical differences between the time points of the heat tolerance test and the participants with and without a history of EHS
